# *Aedes aegypti Ir8a* mutant female mosquitoes show increased attraction to standing water

**DOI:** 10.1080/19420889.2019.1681063

**Published:** 2019-10-25

**Authors:** Joshua I. Raji, Sheyla Gonzalez, Matthew DeGennaro

**Affiliations:** Department of Biological Sciences & Biomolecular Sciences Institute, Florida International University, Miami, FL, USA

**Keywords:** IR8a, water, hygrosensation, mutant, mosquito, *Aedes aegypti*

## Abstract

The detection of water sources is crucial for insects such as mosquitoes to avoid desiccation and survive. In addition, mosquitoes use humidity cues to successfully navigate the environment to find a suitable oviposition site. Previous studies have implicated some members of the ionotropic receptor family in humidity sensing by *Drosophila*. Here, we investigate if IR8a co-receptor mediates water detection in *Aedes aegypti* mosquitoes. Using a simple behavioral assay, we examined the attraction of *Ir8a* mutant mosquitoes to standing water. *Ir8a* mutant mosquitoes were able to discriminate between traps containing water and those without as well as wild-type and heterozygous control females. Surprisingly, the female mutants were more robustly drawn to standing water than control mosquitoes. Further investigation revealed that the increased behavioral attraction to water is likely not mediated by a metabolic need or an activity defect.

The ability to sense water in the environment, hygrosensation, has been previously studied in a number of insect species [1–4]. The availability of water impacts insect longevity, fitness and geographic distribution [6]. Although insects are covered in chitinous exoskeleton, they constantly experience water loss via the cuticle and through their open respiratory systems [7]. Insects with large surface area to volume ratios such as mosquitoes must figure out a way to replenish water loss and maintain internal osmotic balance. Water vapor emanating from oviposition sites have been shown to elicit pre-oviposition behavior in *Anopheles gambiae* [3]. Anthropophilic mosquitoes do not only rely on heat, odor and visual cues to find their human hosts [8], the detection of hygrosensory cues has also been proposed to be important during host-seeking [9]. Functional mapping of the pathways that mediate water-seeking behavior could inform how mosquitoes monitor and adjust its hydration state to maintain optimum physiological homeostasis or seek oviposition sites. Understanding the molecular basis of mosquito water-seeking behavior could lead to novel approaches for controlling mosquito populations and manipulating gravid female attraction to water-baited traps.

Insects possess two distinct systems for detecting water sources: the gustatory system, which is tuned to sensing liquid water [10,11], and the hygrosensory system required for detecting water vapor [4,5]. In *Drosophila*, behavioral response to liquid water was disrupted by ablating the *ppk28* gene function which labels the gustatory water sensory neurons [11]. The gustatory system was activated only after the sensory neurons had made direct contact with liquid water. This draws attention to the hygrosensory system that detects water vapor from a distance. The TRP channels, *nan* and *wtrw*, previously identified in *Drosophila* mediate two contrasting behavioral responses to dry air and moist air respectively [12]. Studies have shown the existence of water sensitive receptors expressed in the coeloconic sensilla of the *Drosophila* antennae [13]. Some ionotropic receptors (IRs) including IR25a, IR93a, IR68a and IR40a which are expressed in these sensilla have been reported to mediate humidity sensing in *Drosophila* [4,5,14].

We recently reported that the *Ir8a* gene (AAEL002922) is key for yellow fever mosquitoes to detect acids in human sweat [15]. Here, we asked if the IR8a pathway also drives water-seeking behavior. To test this, we presented two ramekins (3.8cm height by 5cm width) housed in a trap (16cm height, 9cm width, 6cm diameter) and set at an angle 45° opposite and placed 3.7cm apart from each other inside a rearing cage (30cm height, 28cm diameter, 15cm diameter). One of the ramekins contained 25ml deionized water whereas the other was left blank (Figure 1(a–d)). A total of 50 mosquitoes aged 7–10 days old, previously starved on water for 24hr were introduced into the cage. The assay lasted for 15hrs (27°C, 40% RH) under a 14:10 light-dark cycle (lights on at 8 am).

**Figure 1.**
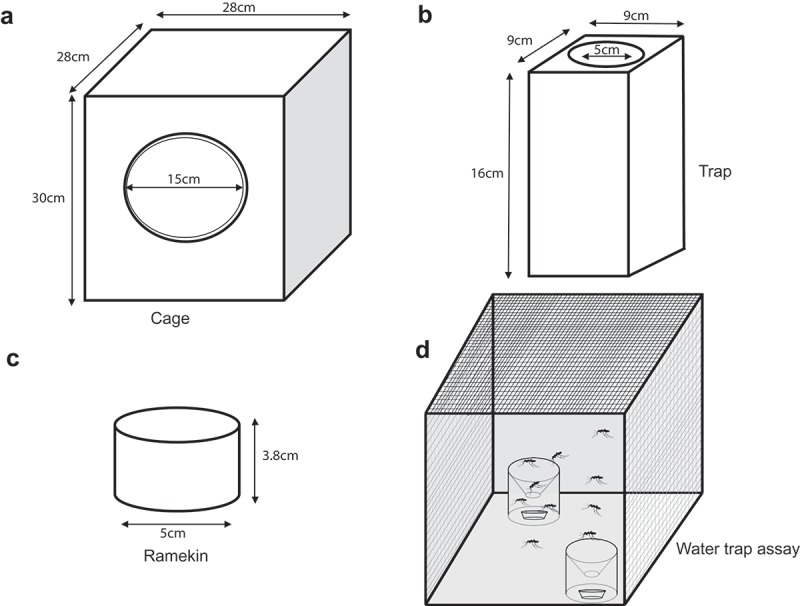
**Mosquito water trap assay**. (a) Illustrations showing the dimensions of a mosquito cage, (b) trap, and (c). ramekin used for the water trap assay (d) Illustration showing ramekin housed in a trap and set at an angle 45° opposite each other and 3.7cm apart. One of the ramekins contained 25ml deionized water whereas the other was left blank.

Thereafter, mosquitoes were cold anesthetized at 4^ο^C for 30 mins. The number of mosquitoes inside each trap was visually scored. To control for possible position effects, the ramekins containing water was swapped after each trial. Surprisingly, *Ir8a* mutant female mosquitoes were more strongly attracted to the water trap than the wild-type and heterozygous controls (Figure 2(a)). This sexually dimorphic phenotype was observed in the *Ir8a* mutant females but not males (Figure 2(b)). All the genotypes tested including males and females show strong preference for water trap over the blank one (Figure 2(c,d)). This suggests that *Ir8a* is not required to find standing water, but rather it influences the intensity of the response to water.

**Figure 2.**
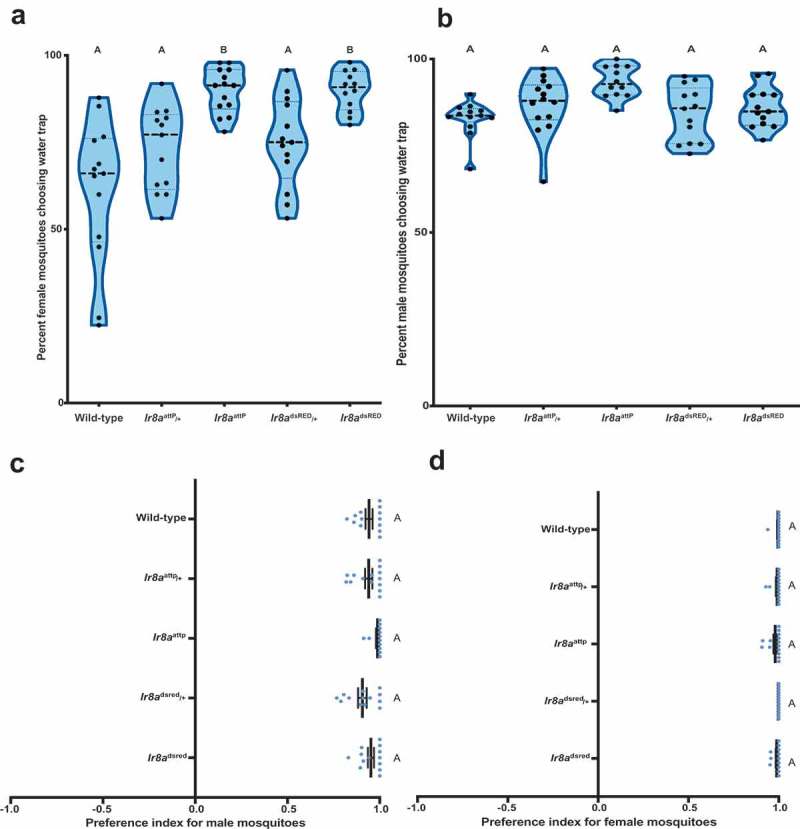
***Ir8a* mutant female mosquitoes are more attracted to water** (a) Response of female mosquitoes and (b) male mosquitoes to water over a period of 15 hours. Genotypes varied in their response to water. Females (one-way ANOVA, *p* <0.0001 n = 12–14). Males (one-way ANOVA *p* =0.0023, n = 12–14). (c) Figure showing female (one-way ANOVA, *p* = 0.213, n = 12–14) and (d) male mosquitoes’ preference toward water source (one-way ANOVA, *p* = 0.055, n = 12–14). Genotypes marked with different letters are significantly different by post hoc Tukey’s HSD test. On the violin plot, the central line represents the median. The shape of the kernel represents the density of the population. Wider sections of the violin plot represent a higher probability that members of the population will fall within the section.

We asked if the strong attraction to water recorded in *Ir8a* mutant females is due to physiological need for hydration or carbohydrates. Using the capillary feeder assay (CAFE) as previously described [16], we quantified the amount of water ingested after 2hrs, and compared to the wild-type and heterozygous controls. Interestingly, we found no significant difference in the total volume of water ingested by *Ir8a* mutants when compared to the wild-type and heterozygous controls (Figure 3(a)). We then wondered if *Ir8a* mutant females are more attracted to the water source because they are looking for a sugar meal. A water solution containing 10% sucrose was presented to the mosquitoes using the CAFE assay. After 4hrs of *ad libitum* feeding, we could not record any feeding difference between the wild-type and *Ir8a* mutant females (Figure 3(b)) similar to what has been previously reported for 18 hours of feeding [15]. Taken together, these findings suggest that the increased attraction to water seen in *Ir8a* mutants cannot be explained by thirst or lack of carbohydrate reserves. However, the resolution of these assays is limited. It is still formally possible that there is a minor defect in water or sucrose consumption due to the loss of *Ir8a* and this could lead to increased sensitivity to water loss.

**Figure 3.**
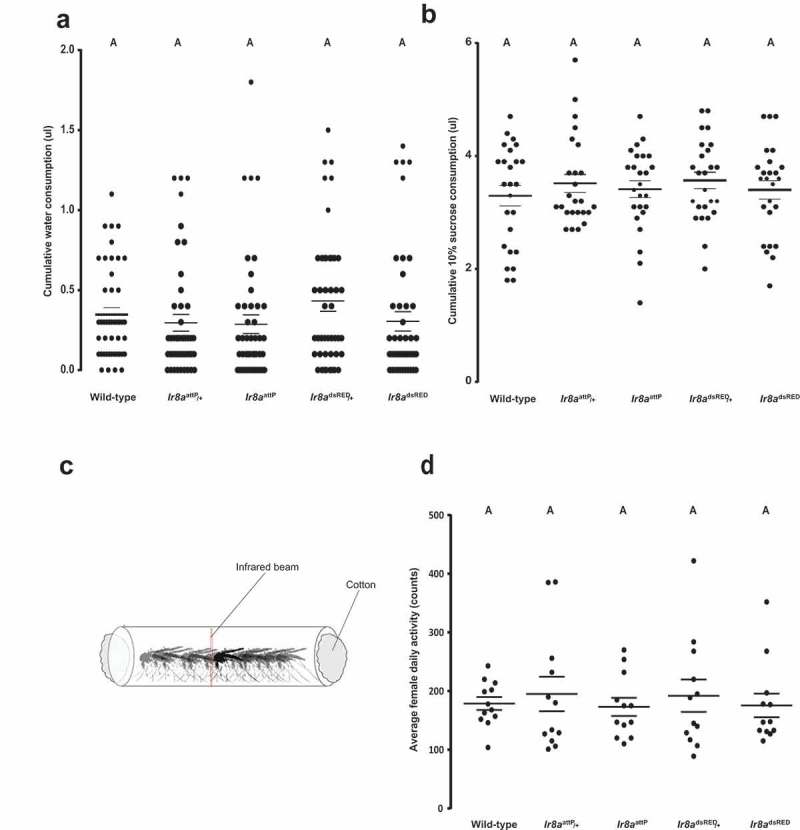
***Ir8a* mutant female mosquitoes feed normally on water and sugar as well as show normal activity**. (a)Water ingestion quantified after 2hrs (one-way ANOVA *p* = 0.3525, n = 45) of drinking. (b) Sucrose consumption recorded after 4hrs of feeding, (one-way ANOVA *p =* 0.7772, n = 25). (c) Illustration of the beam break assay. The red line represents the infrared beam triggered by mosquito movement in the glass tube. An activity count is recorded when a mosquito moves past the beam. (d) Average daily locomotor activity after 4 days of fasting on water, measured by the number of infrared beam breaks (counts). There were no statistical differences among genotypes (p = 0.9320, n=12). On the dot plots, long lines represent the mean and short lines represent standard error. Data was analyzed by one-way ANOVA, and genotypes marked with the same letters are not significantly different by post hoc Tukey’s HSD test.

We next investigated if *Ir8a* mutant females have increased activity in humid environments. We previously reported that the IR8a pathway does not regulate mosquito activity [15]. In the previous study, the glass tube was plugged with a dry and a water-saturated cotton at opposite ends to create a humidity gradient. We reasoned that water saturating both cottons would simulate a wet environment that favors the *Ir8a* mutants, and we could record higher infrared beam breaks triggered by the water-seeking behavior of the mutants. Using a locomotor activity assay adapted to mosquitoes (Figure 3(c)) [17], we found no difference in activity in the *Ir8a* mutants when compared to the wild-type and heterozygous controls (Figure 3(d)). The results were similar to when the assay was performed with only one water-saturated cotton ball [15]. Since *Ir8a* mutants retained normal activity in a humid environment, this does not support the explanation that the increased attraction of *Ir8a* mutants to standing water is due to increased locomotor activity in the presence of increased humidity.

Taken together, the robust water-seeking behavior recorded in the *Ir8a* mutant female mosquitoes is unlikely to be explained by an increased physiological need for water or sugar, but this cannot be entirely ruled out by the data presented here. Also, the mutants show no locomotor activity difference from controls. We propose that the strong attraction to standing water could be mediated by chemosensation. A possibility is that *Ir8a* mutant females might be compensating for the loss of IR8a-dependent olfactory sensory input that has been shown to mediate host-seeking by priming the sensory system toward other vital resources key for survival. It has been previously shown that one sensory input may cause enhanced sensitivity to a completely different sense [18]. Another hypothesis is that *Ir8a* could be part of a neural circuit that represses water-sensing. *Ir8a* may also be important for finding a suitable habitat that is less humid. Future study is needed to uncover the exact contribution of the *Ae. aegypti* IR8a pathway in female mosquito water-seeking behavior.
